# 2-(1*H*-Benzimidazol-1-yl)-1-phenyl­ethanone

**DOI:** 10.1107/S1600536808019107

**Published:** 2008-06-28

**Authors:** Özden Özel Güven, Taner Erdoğan, Simon J. Coles, Tuncer Hökelek

**Affiliations:** a Zonguldak Karaelmas University, Department of Chemistry, 67100 Zonguldak, Turkey; b Southampton University, Department of Chemistry, Southampton SO17 1BJ, England; c Hacettepe University, Department of Physics, 06800 Beytepe, Ankara, Turkey

## Abstract

In the mol­ecule of the title compound, C_15_H_12_N_2_O, the planar benzimidazole system is oriented at a dihedral angle of 80.43 (5)° with respect to the phenyl ring. In the crystal structure, non-classical inter­molecular C—H⋯N and C—H⋯O hydrogen bonds link the mol­ecules into layers parallel to the *ab* plane.

## Related literature

For general backgroud, see: Göker *et al.* (2002 ▶); Özden *et al.* (2004 ▶); Özel Güven *et al.* (2007*a* ▶,*b* ▶); Schar *et al.* (1976 ▶). For related literature, see: Peeters *et al.* (1997 ▶); Freer *et al.* (1986 ▶); Özel Güven *et al.* (2007 ▶).
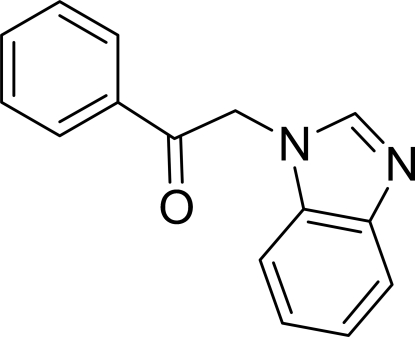


## Experimental

### 

#### Crystal data

C_15_H_12_N_2_O
*M* *_r_* = 236.27
Monoclinic, 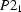

*a* = 5.0475 (2) Å
*b* = 11.2319 (6) Å
*c* = 10.3517 (5) Å
β = 96.620 (3)°
*V* = 582.96 (5) Å^3^
*Z* = 2
Mo *K*α radiation
μ = 0.09 mm^−1^
*T* = 120 (2) K
0.45 × 0.22 × 0.03 mm

#### Data collection

Bruker–Nonius KappaCCD diffractometer
Absorption correction: multi-scan (*SADABS*; Sheldrick, 2007 ▶) *T*_min_ = 0.962, *T*_max_ = 0.997
6918 measured reflections
2639 independent reflections
2265 reflections with *I* > 2σ(*I*)
*R*_int_ = 0.057

#### Refinement

*R*[*F* ^2^ > 2σ(*F* ^2^)] = 0.047
*wR*(*F* ^2^) = 0.119
*S* = 1.06
2639 reflections
212 parameters
1 restraint
All H-atom parameters refined
Δρ_max_ = 0.23 e Å^−3^
Δρ_min_ = −0.22 e Å^−3^

### 

Data collection: *COLLECT* (Hooft, 1998 ▶); cell refinement: *DENZO* (Otwinowski & Minor, 1997 ▶) and *COLLECT*; data reduction: *DENZO* and *COLLECT*; program(s) used to solve structure: *SHELXS97* (Sheldrick, 2008 ▶); program(s) used to refine structure: *SHELXL97* (Sheldrick, 2008 ▶); molecular graphics: *ORTEP-3 for Windows* (Farrugia, 1997 ▶); software used to prepare material for publication: *WinGX* (Farrugia, 1999 ▶).

## Supplementary Material

Crystal structure: contains datablocks I, global. DOI: 10.1107/S1600536808019107/si2097sup1.cif

Structure factors: contains datablocks I. DOI: 10.1107/S1600536808019107/si2097Isup2.hkl

Additional supplementary materials:  crystallographic information; 3D view; checkCIF report

## Figures and Tables

**Table 1:** Hydrogen-bond geometry (Å, °)

*D*—H⋯*A*	*D*—H	H⋯*A*	*D*⋯*A*	*D*—H⋯*A*
C8—H81⋯N2^i^	0.95 (2)	2.43 (2)	3.355 (3)	165.2 (17)
C8—H82⋯O1^ii^	0.98 (2)	2.38 (2)	3.351 (3)	170.1 (17)

Symmetry codes: (i) 
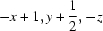
; (ii) 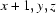.

**Fig. 1.**
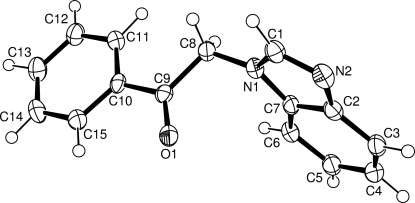
The molecular structure of the title molecule with the atom-numbering scheme. Displacement ellipsoids are drawn at the 50% probability level.

**Fig. 2.**
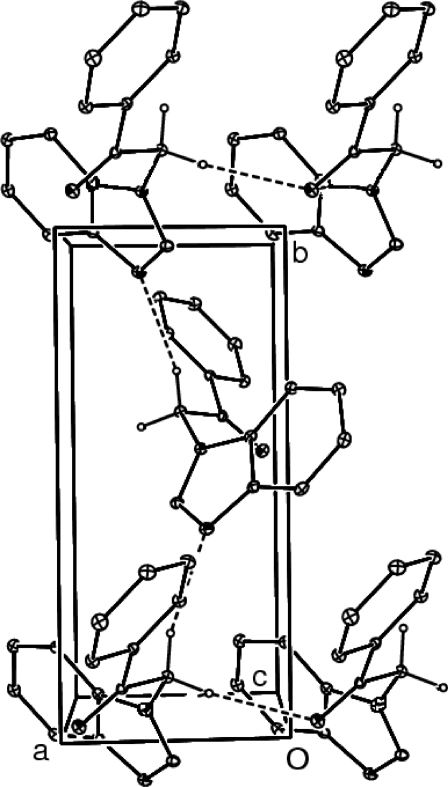
A partial packing diagram of the title compound, viewed along the c axis. Hydrogen bonds are shown as dashed lines. H atoms not involved in hydrogen bonding have been omitted for clarity.

## supplementary crystallographic information

### Comment

Benzimidazoles have been shown to exhibit a large number of biological activities. Some of the substituted benzimidazole derivatives have highly potent antifungal (Göker *et al.*, 2002) and antibacterial (Özden *et al.*, 2004) activities. Recently, it has been reported that benzimidazole ring containing aryl ethers (Özel Güven *et al.*, 2007*a*,b) similar to miconazole (Peeters *et al.*, 1997) and econazole (Freer *et al.*, 1986) structures have more antibacterial activities than antifungal activities (Schar *et al.*, 1976). The crystal structure of oxime form of the benzimidazole substituted ketone has been reported previously (Özel Güven *et al.*, 2007). We report herein the crystal structure of ketone, which is a starting material for biologically important molecules.

In the molecule of the title compound (Fig. 1) the bond lengths and angles are generally within normal ranges. The planar benzimidazole ring system is oriented with respect to the phenyl ring at a dihedral angle of 80.43 (5)°. Atoms C8 and C9 are -0.010 (2) Å and 0.044 (2) Å away from the ring planes of benzimidazole and phenyl, respectively. So, they are coplanar with the adjacent rings. The N1—C8—C9 [111.88 (15)°] and C8—C9—C10 [117.10 (15)°] bond angles are highly different from each other, while O1—C9—C8 [121.07 (16)°] and O1—C9—C10 [121.84 (17)°] bond angles are nearly equal. The N1—C1—N2 [114.25 (18)°], N2—C2—C7 [110.35 (17)°] and C2—C7—C6 [123.30 (18)°] bond angles are enlarged, while C5—C6—C7 [116.34 (19)°] bond angle is narrowed, probably due to the intermolecular C—H···N and C—H···O hydrogen bonds (Table 1).

In the crystal structure, non-classical intermolecular C—H···N and C—H···O hydrogen bonds (Table 1) link the molecules into layers parallel to the *a*,*b* plane (Fig. 2), in which they seem to be effective in the stabilization of the crystal structure.

### Experimental

The title compound, was synthesized by the reaction of 2-bromo-1-phenyl- ethanone (Özel Güven *et al.*, 2007*a*) with 1*H*-benzimidazole. A solution of 2-bromo-1-phenylethanone (4.00 g, 20.10 mmol) in dioxane-ether (8 ml) was added to an ice-cold solution of benzimidazole (11.87 g, 100.5 mmol) in methanol (20 ml) over 60 min under argon atmosphere. The reaction mixture was warmed to ambient temperature and stirred for an additional 18 h, diluted with water (20 ml), and then extracted with chloroform. Organic extract was dried over anhydrous sodium sulfate, concentrated under reduced pressure, and then chromatographed on neutral silica-gel column using chloroform-methanol as eluent. Crystals suitable for X-ray analysis were obtained by the recrystallization of the ketone from a mixture of hexane/ethyl acetate (1:2) (yield; 2.85 g, 60%).

### Refinement

In the absence of significant anomalous scattering effects, Friedel pairs were merged. H atoms were located in difference syntheses and refined isotropically [C—H = 0.95 (2)–1.06 (3) Å; *U*_iso_(H) = 0.020 (5)–0.061 (9) Å^2^].

### Crystal data

**Table:**

C_15_H_12_N_2_O	*F*_000_ = 248
*M**_r_* = 236.27	*D*_x_ = 1.346 Mg m^−^^3^
Monoclinic, *P*2_1_	Mo *K*α radiation λ = 0.71073 Å
Hall symbol: P 2yb	Cell parameters from 1379 reflections
*a* = 5.0475 (2) Å	θ = 2.9–27.5º
*b* = 11.2319 (6) Å	µ = 0.09 mm^−^^1^
*c* = 10.3517 (5) Å	*T* = 120 (2) K
β = 96.620 (3)º	Plate, colorless
*V* = 582.96 (5) Å^3^	0.45 × 0.22 × 0.03 mm
*Z* = 2	

### Data collection

**Table:**

Bruker–Nonius Roper CCD camera on κ-goniostat diffractometer	2639 independent reflections
Radiation source: fine-focus sealed tube	2265 reflections with *I* > 2σ(*I*)
Monochromator: graphite	*R*_int_ = 0.057
Detector resolution: 9.091 pixels mm^-1^	θ_max_ = 27.5º
*T* = 120(2) K	θ_min_ = 3.6º
φ and ω scans	*h* = −6→6
Absorption correction: multi-scan(SADABS; Sheldrick, 2007)	*k* = −14→14
*T*_min_ = 0.962, *T*_max_ = 0.997	*l* = −13→13
6918 measured reflections	

### Refinement

**Table:**

Refinement on *F*^2^	Secondary atom site location: difference Fourier map
Least-squares matrix: full	Hydrogen site location: inferred from neighbouring sites
*R*[*F*^2^ > 2σ(*F*^2^)] = 0.047	All H-atom parameters refined
*wR*(*F*^2^) = 0.119	*w* = 1/[σ^2^(*F*_o_^2^) + (0.0651*P*)^2^ + 0.019*P*] where *P* = (*F*_o_^2^ + 2*F*_c_^2^)/3
*S* = 1.06	(Δ/σ)_max_ < 0.001
2639 reflections	Δρ_max_ = 0.23 e Å^−^^3^
212 parameters	Δρ_min_ = −0.22 e Å^−^^3^
1 restraint	Extinction correction: SHELXL97 (Sheldrick, 2008), Fc^*^=kFc[1+0.001xFc^2^λ^3^/sin(2θ)]^-1/4^
Primary atom site location: structure-invariant direct methods	Extinction coefficient: 0.091 (14)

### Special details

**Table:**

Geometry. All e.s.d.'s (except the e.s.d. in the dihedral angle between two l.s. planes) are estimated using the full covariance matrix. The cell e.s.d.'s are taken into account individually in the estimation of e.s.d.'s in distances, angles and torsion angles; correlations between e.s.d.'s in cell parameters are only used when they are defined by crystal symmetry. An approximate (isotropic) treatment of cell e.s.d.'s is used for estimating e.s.d.'s involving l.s. planes.
Refinement. Refinement of *F*^2^ against ALL reflections. The weighted *R*-factor *wR* and goodness of fit *S* are based on *F*^2^, conventional *R*-factors *R* are based on *F*, with *F* set to zero for negative *F*^2^. The threshold expression of *F*^2^ > σ(*F*^2^) is used only for calculating *R*-factors(gt) *etc*. and is not relevant to the choice of reflections for refinement. *R*-factors based on *F*^2^ are statistically about twice as large as those based on *F*, and *R*- factors based on ALL data will be even larger.

### Fractional atomic coordinates and isotropic or equivalent isotropic displacement parameters (Å^2^)

**Table:**

	*x*	*y*	*z*	*U*_iso_*/*U*_eq_	
O1	0.0968 (3)	0.55671 (14)	0.26162 (13)	0.0288 (4)	
N1	0.3784 (3)	0.56655 (14)	0.05460 (15)	0.0236 (4)	
N2	0.3592 (4)	0.41520 (16)	−0.08838 (17)	0.0301 (4)	
C1	0.4848 (4)	0.46224 (19)	0.0180 (2)	0.0265 (4)	
H1	0.651 (6)	0.432 (3)	0.074 (3)	0.048 (7)*	
C2	0.1553 (4)	0.49603 (17)	−0.12561 (19)	0.0251 (4)	
C3	−0.0424 (4)	0.49415 (19)	−0.2318 (2)	0.0288 (5)	
H3	−0.051 (5)	0.422 (2)	−0.291 (2)	0.034 (6)*	
C4	−0.2249 (4)	0.5857 (2)	−0.2443 (2)	0.0315 (5)	
H4	−0.366 (5)	0.584 (3)	−0.317 (3)	0.045 (7)*	
C5	−0.2133 (4)	0.67987 (19)	−0.1537 (2)	0.0293 (5)	
H5	−0.340 (4)	0.742 (2)	−0.168 (2)	0.020 (5)*	
C6	−0.0171 (4)	0.68387 (18)	−0.0477 (2)	0.0258 (4)	
H6	−0.013 (5)	0.753 (3)	0.011 (3)	0.048 (7)*	
C7	0.1634 (4)	0.59090 (17)	−0.03658 (18)	0.0237 (4)	
C8	0.4659 (4)	0.63860 (18)	0.16674 (18)	0.0241 (4)	
H81	0.481 (4)	0.720 (2)	0.143 (2)	0.025 (5)*	
H82	0.642 (4)	0.609 (2)	0.203 (2)	0.025 (5)*	
C9	0.2788 (4)	0.62815 (17)	0.27150 (18)	0.0222 (4)	
C10	0.3283 (4)	0.70936 (18)	0.38542 (18)	0.0226 (4)	
C11	0.5216 (4)	0.79730 (19)	0.39188 (18)	0.0273 (5)	
H11	0.646 (4)	0.805 (2)	0.326 (2)	0.024 (5)*	
C12	0.5582 (4)	0.8738 (2)	0.4985 (2)	0.0310 (5)	
H12	0.695 (5)	0.934 (2)	0.503 (2)	0.031 (6)*	
C13	0.3975 (4)	0.8617 (2)	0.5977 (2)	0.0351 (5)	
H13	0.420 (5)	0.920 (3)	0.678 (3)	0.047 (7)*	
C14	0.2068 (5)	0.7743 (2)	0.5926 (2)	0.0352 (5)	
H14	0.089 (6)	0.761 (3)	0.658 (3)	0.061 (9)*	
C15	0.1691 (4)	0.69726 (19)	0.4872 (2)	0.0284 (5)	
H15	0.036 (4)	0.636 (2)	0.481 (2)	0.025 (6)*	

### Atomic displacement parameters (Å^2^)

**Table:**

	*U*^11^	*U*^22^	*U*^33^	*U*^12^	*U*^13^	*U*^23^
O1	0.0307 (7)	0.0341 (8)	0.0215 (7)	−0.0053 (6)	0.0026 (5)	0.0005 (6)
N1	0.0279 (8)	0.0236 (8)	0.0200 (8)	−0.0005 (7)	0.0049 (6)	−0.0003 (6)
N2	0.0377 (9)	0.0257 (8)	0.0282 (9)	−0.0016 (8)	0.0098 (7)	−0.0009 (7)
C1	0.0316 (10)	0.0239 (9)	0.0249 (10)	−0.0013 (8)	0.0071 (8)	0.0010 (8)
C2	0.0276 (9)	0.0245 (10)	0.0245 (10)	−0.0034 (8)	0.0085 (7)	−0.0015 (8)
C3	0.0341 (10)	0.0313 (11)	0.0219 (10)	−0.0098 (9)	0.0075 (8)	−0.0055 (8)
C4	0.0300 (11)	0.0417 (12)	0.0230 (10)	−0.0051 (9)	0.0034 (8)	0.0013 (8)
C5	0.0303 (10)	0.0336 (12)	0.0244 (10)	0.0019 (9)	0.0052 (8)	0.0009 (8)
C6	0.0297 (10)	0.0265 (10)	0.0222 (9)	0.0016 (8)	0.0072 (7)	−0.0015 (8)
C7	0.0271 (9)	0.0276 (10)	0.0170 (9)	−0.0050 (8)	0.0045 (7)	0.0006 (8)
C8	0.0269 (10)	0.0256 (11)	0.0200 (9)	−0.0007 (8)	0.0029 (8)	−0.0002 (8)
C9	0.0210 (9)	0.0241 (9)	0.0207 (9)	0.0012 (8)	−0.0003 (7)	0.0034 (7)
C10	0.0249 (9)	0.0246 (9)	0.0173 (9)	0.0043 (8)	−0.0022 (7)	0.0027 (7)
C11	0.0293 (10)	0.0311 (11)	0.0218 (10)	−0.0011 (9)	0.0041 (8)	0.0027 (8)
C12	0.0378 (11)	0.0294 (11)	0.0248 (10)	−0.0045 (10)	−0.0001 (9)	−0.0017 (8)
C13	0.0464 (13)	0.0341 (12)	0.0248 (11)	−0.0012 (10)	0.0043 (9)	−0.0040 (9)
C14	0.0414 (11)	0.0437 (13)	0.0222 (10)	−0.0004 (10)	0.0101 (9)	−0.0020 (9)
C15	0.0297 (10)	0.0311 (11)	0.0243 (10)	−0.0029 (9)	0.0032 (7)	0.0015 (8)

### Geometric parameters (Å, °)

**Table:**

O1—C9	1.215 (2)	C7—C2	1.406 (3)
N1—C1	1.361 (3)	C8—H81	0.95 (3)
N1—C7	1.381 (2)	C8—H82	0.98 (2)
N1—C8	1.442 (3)	C9—C8	1.522 (3)
N2—C1	1.317 (3)	C10—C9	1.489 (3)
N2—C2	1.393 (3)	C10—C15	1.403 (3)
C1—H1	1.02 (3)	C11—C10	1.384 (3)
C3—C2	1.398 (3)	C11—C12	1.394 (3)
C3—C4	1.377 (3)	C11—H11	0.99 (2)
C3—H3	1.01 (3)	C12—H12	0.96 (3)
C4—H4	0.98 (3)	C13—C12	1.386 (3)
C5—C4	1.410 (3)	C13—C14	1.371 (3)
C5—H5	0.95 (2)	C13—H13	1.06 (3)
C6—C5	1.391 (3)	C14—C15	1.389 (3)
C6—C7	1.382 (3)	C14—H14	0.96 (3)
C6—H6	0.99 (3)	C15—H15	0.96 (2)
			
C1—N1—C7	106.55 (16)	N1—C8—H81	111.1 (13)
C1—N1—C8	127.82 (17)	N1—C8—H82	107.2 (13)
C7—N1—C8	125.63 (16)	C9—C8—H81	109.4 (13)
C1—N2—C2	103.83 (17)	C9—C8—H82	108.0 (13)
N1—C1—H1	116.9 (16)	H81—C8—H82	109.1 (19)
N2—C1—N1	114.25 (18)	O1—C9—C8	121.07 (16)
N2—C1—H1	128.8 (16)	O1—C9—C10	121.84 (17)
N2—C2—C3	130.22 (18)	C10—C9—C8	117.10 (15)
N2—C2—C7	110.35 (17)	C11—C10—C9	121.91 (17)
C3—C2—C7	119.43 (18)	C11—C10—C15	119.61 (18)
C2—C3—H3	117.7 (14)	C15—C10—C9	118.47 (18)
C4—C3—C2	118.14 (19)	C10—C11—C12	120.42 (18)
C4—C3—H3	124.0 (14)	C10—C11—H11	122.1 (13)
C3—C4—C5	121.5 (2)	C12—C11—H11	117.4 (13)
C3—C4—H4	118.4 (17)	C13—C12—C11	119.4 (2)
C5—C4—H4	120.1 (17)	C13—C12—H12	120.8 (15)
C6—C5—C4	121.3 (2)	C11—C12—H12	119.8 (15)
C6—C5—H5	120.4 (13)	C12—C13—H13	120.3 (15)
C4—C5—H5	118.3 (13)	C14—C13—C12	120.7 (2)
C5—C6—H6	118.1 (15)	C14—C13—H13	119.0 (15)
C7—C6—C5	116.34 (19)	C13—C14—C15	120.5 (2)
C7—C6—H6	125.5 (15)	C13—C14—H14	125 (2)
N1—C7—C2	105.01 (16)	C15—C14—H14	115 (2)
N1—C7—C6	131.69 (17)	C10—C15—H15	118.6 (13)
C6—C7—C2	123.30 (18)	C14—C15—C10	119.4 (2)
N1—C8—C9	111.88 (15)	C14—C15—H15	122.0 (13)
			
C7—N1—C1—N2	−0.3 (2)	N1—C7—C2—N2	0.66 (19)
C8—N1—C1—N2	178.87 (18)	N1—C7—C2—C3	−179.80 (17)
C1—N1—C7—C2	−0.21 (18)	C6—C7—C2—N2	−179.13 (17)
C1—N1—C7—C6	179.6 (2)	C6—C7—C2—C3	0.4 (3)
C8—N1—C7—C2	−179.44 (17)	O1—C9—C8—N1	7.0 (2)
C8—N1—C7—C6	0.3 (3)	C10—C9—C8—N1	−172.76 (16)
C1—N1—C8—C9	−105.9 (2)	C11—C10—C9—O1	−174.63 (18)
C7—N1—C8—C9	73.2 (2)	C11—C10—C9—C8	5.1 (3)
C2—N2—C1—N1	0.7 (2)	C15—C10—C9—O1	3.9 (3)
C1—N2—C2—C3	179.7 (2)	C15—C10—C9—C8	−176.38 (17)
C1—N2—C2—C7	−0.8 (2)	C9—C10—C15—C14	−178.14 (19)
C4—C3—C2—N2	178.8 (2)	C11—C10—C15—C14	0.4 (3)
C4—C3—C2—C7	−0.7 (3)	C10—C11—C12—C13	−0.6 (3)
C2—C3—C4—C5	0.6 (3)	C12—C11—C10—C9	178.37 (18)
C6—C5—C4—C3	−0.2 (3)	C12—C11—C10—C15	−0.1 (3)
C5—C6—C7—N1	−179.7 (2)	C14—C13—C12—C11	1.1 (3)
C5—C6—C7—C2	0.0 (3)	C12—C13—C14—C15	−0.8 (3)
C7—C6—C5—C4	−0.1 (3)	C13—C14—C15—C10	0.0 (3)

### Hydrogen-bond geometry (Å, °)

**Table:**

*D*—H···*A*	*D*—H	H···*A*	*D*···*A*	*D*—H···*A*
C8—H81···N2^i^	0.95 (2)	2.43 (2)	3.355 (3)	165.2 (17)
C8—H82···O1^ii^	0.98 (2)	2.38 (2)	3.351 (3)	170.1 (17)

Symmetry codes: (i) −*x*+1, *y*+1/2, −*z*; (ii) *x*+1, *y*, *z*.

## References

[bb1] Farrugia, L. J. (1997). *J. Appl. Cryst.* **30**, 565.

[bb2] Farrugia, L. J. (1999). *J. Appl. Cryst.* **32**, 837–838.

[bb3] Freer, A. A., Pearson, A. & Salole, E. G. (1986). *Acta Cryst.* C**42**, 1350–1352.

[bb4] Göker, H., Kuş, C., Boykin, D. W., Yıldız, S. & Altanlar, N. (2002). *Bioorg. Med. Chem.* **10**, 2589–2596. 10.1016/s0968-0896(02)00103-7 12057648

[bb5] Hooft, R. W. W. (1998). *COLLECT* Nonius BV, Delft, The Netherlands.

[bb6] Otwinowski, Z. & Minor, W. (1997). *Methods in Enzymology*, Vol. 276, *Macromolecular Crystallography*, Part A, edited by C. W. Carter Jr & R. M. Sweet, pp. 307–326. New York: Academic Press.

[bb7] Özden, S., Karataş, H., Yıldız, S. & Göker, H. (2004). *Arch. Pharm.* **337**, 556–562. 10.1002/ardp.200400884 15476288

[bb8] Özel Güven, Ö. el, Erdoğan, T., Çaylak, N. & Hökelek, T. (2007). *Acta Cryst.* E**63**, o3463–o3464.

[bb9] Özel Güven, Ö., Erdoğan, T., Göker, H. & Yıldız, S. (2007*a*). *Bioorg. Med. Chem. Lett.* **17**, 2233–2236. 10.1016/j.bmcl.2007.01.061 17289382

[bb10] Özel Güven, Ö., Erdoğan, T., Göker, H. & Yıldız, S. (2007*b*). *J. Heterocycl. Chem.* **44**, 731–734.

[bb11] Peeters, O. M., Blaton, N. M. & De Ranter, C. J. (1997). *Bull. Soc. Chim. Belg.* **88**, 265–272.

[bb12] Schar, G., Kayser, F. H. & Dupont, M. C. (1976). *Chemotheraphy*, **22**, 211–220. 10.1159/000221928 817875

[bb13] Sheldrick, G. M. (2007). *SADABS* Bruker AXS Inc., Madison, Wisconsin, USA.

[bb14] Sheldrick, G. M. (2008). *Acta Cryst.* A**64**, 112–122. 10.1107/S0108767307043930 18156677

